# Revisiting the dimensional structure of the Edinburgh Postnatal Depression Scale (EPDS): empirical evidence for a general factor

**DOI:** 10.1186/1471-2288-11-93

**Published:** 2011-06-20

**Authors:** Michael E Reichenheim, Claudia L Moraes, Alessandra SD Oliveira, Gustavo Lobato

**Affiliations:** 1 Department of Epidemiology, Institute of Social Medicine (IMS), Rio de Janeiro State University (UERJ), Brazil; 2 Family Health Master Program, Estácio de Sá University, Rio de Janeiro, Brazil; 3 Bezerra de Araújo Faculty (FABA), Rio de Janeiro, Brazil; 4 Fernandes Figueira Institute, Oswaldo Cruz Foundation (FIOCRUZ), Rio de Janeiro, Brazil

## Abstract

**Background:**

The Edinburgh Postnatal Depression Scale (EPDS) has been proposed as a one-dimensional instrument and used as a single 10-item scale. This might be considered questionable since repeated psychometric studies have shown multi-dimensionality, which would entail using separate component subscales. This study reappraised the dimensional structure of the EPDS, with a focus on the extent of factor correlations and related factor-based discriminant validity as a foundation for deciding how to effectively scale the component items.

**Methods:**

The sample comprised 811 randomly selected mothers of children up to 5 months attending primary health services of Rio de Janeiro, Brazil. Strict Confirmatory Factor Analysis (CFA) and Exploratory Factor Analysis modeled within a CFA framework (E/CFA) were sequentially used to identify best fitting and parsimonious model(s), including a bifactor analysis to evaluate the existence of a general factor. Properties concerning the related 10-item raw-score scale were also investigated using non-parametric items response theory methods (scalability and monotonicity).

**Results:**

An initial CFA rejected the one-dimensional structure, while an E/CFA subscribed a three-dimensional solution. Yet, factors were highly correlated (0.66, 0.75 and 0.82). The ensuing CFA showed poor discriminant validity (some square-roots of average variance extracted below the factor correlations). A general bifactor CFA was then fit. Results suggested that, although still weakly encompassing three specific factors, the EPDS might be better described by a model encompassing a general factor (loadings ranging from 0.51 to 0.81). The related 10-item raw score showed adequate scalability (Loevinger's H coefficient = 0.4208), monotonicity e partial double monotonicity (nonintersections of Item Step Response Functions).

**Conclusion:**

Although the EPDS indicated the presence of specific factors, they do not qualify as independent dimensions if used separately and should therefore not be used empirically as sub-scales (raw scores). An all-encompassing scale seems better suited and continuing its use in clinical practice and applied research should be encouraged.

## Background

Post-partum depression (PPD) is a difficult construct to measure in practice [1]. Enabling as many health professionals as possible to make a timely first assessment of maternal mental health while leaving detailed psychiatric assessments for only those situations suggestive of PPD is an appealing approach. Similarly, applied research contexts require swift yet valid instruments. In the late 1980s Cox et al. [2] argued that a suitable instrument to evaluate depressive symptoms after childbirth was needed since available tools to assess depression in general populations put too much emphasis on somatic symptoms that could nevertheless be due to normal physiologic adaptations associated to childbearing. In an attempt to address this drawback, the authors proposed the Edinburgh Postnatal Depression Scale (EPDS), a simple and well accepted 10-item assessment tool that is easy to fill in and does not require specialized psychiatric expertise from health workers. Since its conception, the EPDS has been adapted for use in several countries [3,4] and has become the most widely used instrument for a first approach to PPD [4,5].

The EPDS has been extensively scrutinized and a number of studies have evaluated its psychometric properties. Several studies have focused on its dimensional structure, with at least thirteen comprising sample sizes above 150 individuals [6-18]. Although Cox et al. [2] originally proposed the EPDS as a one-dimensional measurement tool and this has been supported by a few authors [12,13], most of the factorial analyses have shown that the EPDS would be better defined through multi-factorial structures, either by two [6,10,16-18] or three factors [7-9,11,14,15].

Regardless of the number of uncovered factors, most studies clearly distinguished items representing 'anxiety' -- items 3 (*blaming oneself unnecessarily when things [go] wrong*), 4 (*having been anxious or worried for no good reason*) and 5 (*having felt scared or panicky for no very good reason*) -- from those representing low positive affect or anhedonia -- 1 (*[not being] able to laugh and see the funny side of things*) and 2 (*[not] looking forward with enjoyment to things*) -- and depression -- 9 (*feeling unhappy [and] crying*) and 10 ([*thinking] of harming oneself*). In three studies, the items on anhedonia and depression jointly loaded on a single factor forming a two-factorial structure along with an anxiety factor [16-18]. Another study showed several cross-loadings involving items 8 (*feeling sad or miserable*), 9 and 10 [10]. A fifth study suggesting a two-factor structure was less conclusive since a few items had been removed before the factorial analysis [19].

A distinction between the anxiety items and the others persisted in all studies showing a three-factorial solution, but with items mapping anhedonia and depression now clearly separated. In some studies, item 8 joined items 1 and 2 on anhedonia [8,9,11,14,15], whereas in others this item joined items 9 and 10 on depression [7,13]. Items 6 (*feeling that things have been getting on top of [the respondent]*) and 7 (*having been so unhappy that [respondent] had difficulty sleeping*) showed to be the most ill-behaved irrespectively of the type of solution. They often cross loaded, in some studies joining the anhedonia/depression items and in others grouping with the anxiety items.

Despite some oddities -- which may have come about from methodological shortcomings such as inadequate sample sizes, unsuitable multivariate models (e.g., principal components analysis) and/or failing to correctly model the ordered-categorical nature of the items --, the reviewed literature shows more congruence than otherwise and, without further detailing, would be suggestive of a multi-scale measurement tool. However, considering this alleged dimensional profile in the light of the usual way the instrument has been thus far discussed methodologically and hence used in practice [3,4], a fundamental but yet unanswered question follows. If the EPDS is really multi-dimensional, so far as to suggest an independent anxiety sub-scale [6,18], is it appropriate to use the complete 10-item score and thereafter specify a cut-off point to define PPD as commonly done? From a psychometric stance, in principle, the answer should be 'no' since distinct although not necessarily uncorrelated dimensions of a given construct require separate empirical scales. Therefore, relying on the evidence from the literature, a call for a two- or three-tiered measurement tool would be reasonable.

A connected question concerns the use of raw scores as proxies for latent traits in applied research. Beyond assessing whether these raw scores are effectively matching the purportedly related latent factors scores, asking which factors are actually involved is also necessary. Specifically in regards to the EPDS, should raw scores relate to specific (e.g., anhedonia, anxiety and depression) factors or would a sum of all ten item scores be an adequate representation of an overall dimension covering PPD? It should not go unnoticed that separately using sums of factor-specific items may incur in missing out the full mapping [20] of an overall dimension (in tandem or instead of several specific dimensions), should it exist. Conversely, adding up item scores when items comprise a multidimensional structure would be unwarranted. Different dimensions may also have exclusive antecedents and consequents, which would not be distinguishable if all items were lumped together.

Thus, before accepting a multi-scale usage of an instrument, an important aspect that needs scrutinizing concerns whether there is sufficient discriminatory power across the factors and whether these factors are thus appropriate descriptors of truly independent theoretical dimensions [21]. To the best of the authors' knowledge this scrutiny has never been performed in regards to the EPDS. One purpose of this study was thus to reappraise its dimensional structure with a special focus on the assessment of factor correlations and the related factor-based discriminant validity properties. This evaluation first required a reassessment of the number of constituent factors in order to examine whether the one-dimensional solution originally specified would be again refuted in the present data. Analysis would only proceed if a multi-dimensional structure were supported. Given the hypothesis that the identified factors failed to hold discriminant validity, the identification of a general factor would be further explored through a bifactor analysis [22]. An ensuing objective would then be to scrutinize the properties of raw scores as proper representations of model-based (latent) factor scores.

## Method

### Sample and procedures

Participants were randomly selected mothers of children under 5 months of age waiting to be consulted in five large public primary health care facilities of Rio de Janeiro, Brazil. Data collection took place from January to July 2007. Given a shared research purpose was to study the role of intimate partner violence in the early weaning and/or PPD, women were considered ineligible when experiencing less than 1 month of intimate relationship with partner during pregnancy or the postpartum period. Other exclusion criteria were situations in which there was an absolute contraindication for breastfeeding. Women that gave birth to twins were also excluded, to avoid a very particular and rare subgroup that could not be adequately addressed in the analysis. Out of the 853 women invited to take part in the study, 18 (2.1%) were not eligible and from the remaining 835, 24 (2.9%) refused to participate. Thus, 811 women were effectively interviewed in a reserved area without the presence anyone, but the interviewer, once anonymity and confidentiality of the information collected had been warranted. A Brazilian Portuguese version of the EPDS [23] was completed along with other instruments comprising a comprehensive multidimensional questionnaire.

### Data analysis

**Figure 1:**
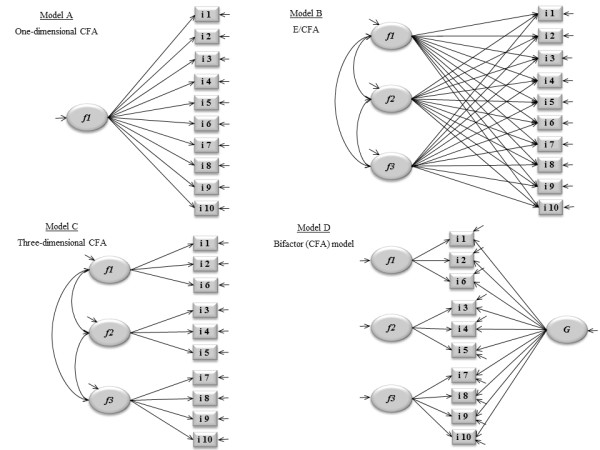
**Path diagrams**. 1A) One-dimensional confirmatory factor model; 1B) Exploratory/confirmatory factor model (testing a three-factor structure); 1C) Three dimensional confirmatory factor model; 1D) Bifactor model - three dimensional confirmatory factor model plus general (g) factor structure.

The dimensional scrutiny began by re-assessing the original one-dimensional structure originally proposed by Cox et al. [2]. To this end a Confirmatory Factor Analysis (CFA) was implemented [21,24] employing Mplus' robust weighted least squares mean and variance adjusted (WLSMV) estimator [25]. The model's diagram is in Figure 1A. Since the EPDS is comprised of ten four-level ordinal items, polychoric correlation matrices were suitably used as automatically generated in Mplus [26]. Goodness of fit (GOF) was evaluated using three indices [21]. The Root Mean Square Error of Approximation (RMSEA) incorporates a penalty function for poor model parsimony [21,27,28]. Values under 0.06 suggest close approximate (adequate) fit, whereas values above 0.10 indicate poor fit and that the model should be rejected [29,30]. The Comparative Fit Index (CFI) and the Tucker-Lewis index (TLI) represent incremental fit indices [21,31] contrasting the hypothesized model to a more restricted nested baseline model, the "null model". Both range from zero to one and values > 0.9 are indicative of adequate fit [21,32].

Anticipating a possible model misfit and/or foreseeing plausible alternative dimensional structures, the next step consisted in re-exploring the dimensional structure of the EPDS through an Exploratory Structural Equation Model procedure as proposed by Marsh et al. [33]. This consisted of fitting a sequence of exploratory models (2 to 4 factors) within a CFA framework (E/CFA). Figure 1B represents a 3-factor E/CFA model. The procedure has the advantage over the 'traditional' Exploratory Factor Analysis (EFA) model [34] for it allows relaxing and effectively implementing several restrictions imposed by an EFA. Freely estimating certain parameters enables testing interesting properties that are otherwise only accomplished with a CFA yet keeping the main gist of an EFA. Notably, all loadings are freely estimated and rotation is possible. The current analysis used the *geomin *oblique rotation [24,30]. Additionally, item residual (error) correlations were also evaluated since conditional dependencies may indicate possible item redundancies [21]. To this end Modification Indices (MI) were used. A MI reflects how much the overall model chi-square decreases if a constrained parameter is freely estimated. Here, possible correlations between item measurement errors involving MI values equal or above 10 would be further examined, as well as the magnitude of the corresponding expected parameter changes (EPC) for freely estimated parameters [21]. The same GOF indices presented before were used here too. Theoretical meaningfulness was also considered to assess the pattern and number of factors.

The next step was to apply a strict CFA-type model to the 'best' E/CFA identified (Figure 1C). Besides reassessing factor loadings and error correlations in a congeneric perspective (i.e., items loading exclusively on purported factors), the sequence also involved assessing factor-based convergent and discriminant validity [21,31]. Both are based on the Average Variance Extracted (AVE) [31]. The AVE assesses the amount of variance captured by a common factor in relation to the amount of variance due to random measurement error [35]. It is a function of the relationship between the standardized item factor loadings and the related measurement error (uniqueness) that refers to the portion of an indicator not explained by the latent factor [36]. Values vary from 0 to 1. A factor shows convergent validity if AVE ≥ 0.50, which is indicative that at least 50% of the variance in a measure is due to the hypothesized underlying trait. Factor-based convergent validity is questionable if AVE < 0.50 since the variance due to measurement error is then greater than the variance due to the construct [35].

In multi-dimensional models, factor-based discriminant validity is said to hold if, for any given factor, the square root of its AVE is above the correlations with any other related factors in the model [37], and preferably without any confidence interval overlap. The 95% CI for AVE and square-root of AVE were obtained via bootstrap method with 1000 replications [38,39]. For reassurance, further interim reassessment of cross loadings and residual correlations were carried out using MI (EPC). The same criteria were applied. Similarly, the fit assessment used the RMSEA, CFI and TLI indices.

Having ascertained proper item-factor specification (absence of relevant cross-loadings), absence of residual correlations, and having identified poor discriminant validity, we engaged in further exploring whether or not the EPDS, as other instruments tapping mood and anxiety disorders and general distress [40-44], would be able to identify a non-specific general (g) factor along with specific ones representing anhedonia, anxiety and depression. To this end, a bifactor modelling procedure was employed [22,45]. The model's diagram is shown in Figure 1D. For identification, all factors were specified as orthogonal [45]. Significance testing (for factors) used the correction procedure proposed by Satorra & Bentler [46], since the difference in chi-square values for two nested models is not chi-square distributed when the WLSMV estimator is used.

Several other properties were inspected in tandem. In order to weigh their relative importance, the percentages of variance explained by items, factors (specific and general) and due to errors (uniqueness) were calculated. We also investigated the obtained model-based thresholds [47]. These are equivalent to item response theory (IRT) theta-metric *b_i _*parameters [47] and indicate how items actually map the dimension (latent trait) in terms of increasing intensity or severity. In particular, we looked at how the specific item components varied regarding the g-factor.

We then sought to investigate the role of raw scores as a useful measure to rank respondents along the overall latent trait [48]. Correlations between the generated specific and general factor scores, and the respective total raw scores were first assessed, with a particular eye on the correlation between the g-factor score and the (purportedly equivalent) sum-of-ten-items raw score. Factor scores were estimated using maximum a posteriori method as implemented in Mplus [49].

To complete the process we turned to non-parametric item-response theory (NIRT) methods to look into the properties of the total raw score (*X*_+_, the sum of all 10 EPDS items scores), namely, scalability and monotonicity. Scalability relates to the ability of items and, by extension, the overall *X*_+ _scale to meaningfully order and position subjects along a continuum as their latent trait effectively increases. Scalability was gauged through Loevinger's H coefficient [48] using a special Stata routine [50,51]. As suggested by Mokken, values > 0.3 indicate acceptable levels [48].

Under the Monotone Homogeneity Model, the monotonicity assumption holds when the probability of an item response greater than or equal to any fixed value is a nondecreasing function of the latent trait *θ *[51]. For scales involving polytomous items as the EPDS, by definition, the *m = 3 *item step response functions (ISRF) of any given item containing *m*+1 = 4 levels may not intersect if the monotonicity assumption is sustained [48]. Furthermore, when the double monotonicity assumption holds, besides 'within item' monotonicity (and ensuing nonintersections of the *m *ISRFs), nonintersections also occurs across ISRFs of different items [48]. Under double monotonicity one may be fairly confident that items will be answered and interpreted similarly by all the respondents, whatever their level of the latent trait. Monotonicity (single and double) was evaluated through the criteria proposed by Molenaar, Sijtsma, and Boer [52]. Accordingly, a criterion less than 40 suggests that the reported violations (ISRF intersection) can be ascribed to sampling variation. If the criterion is between 40 and 80, more detailed evaluations are required. A criterion beyond 80 raises doubts about the monotonicity assumption of an item and in turns, about the scale as a whole. Single monotonicity was also inspected graphically by means of the item traces as a function of the restscore. For completeness, the number of violations of monotonicity concerning within-item ISRF intersections, and double monotonicity concerning inter-item ISRF nonintersection was also evaluated. A full account of the methods employed here and details on NIRT may be found in Molenaar, Sijtsma & Boer [52], Sijtsma & Molenaar [48], and Hardouin, Bonnaud-Antignac & Sebille [51].

### Ethics Approval

The study was approved by the Research Ethics Committee of the Rio de Janeiro Municipal Health Department in conformity with the principles embodied in the declaration of Helsinki. A written informed consent was given by study participants after having the informed consent form read to them.

## Results

Mean maternal age was 25.3 years (range 13-44 years, 95% CI: 24.9; 25.8) and 22.7% (95% CI: 19.8; 25.6) of women were adolescents (< 20 years). Most of the participants had steady partners (86.6%, 95% CI: 84.2; 88.9) and up to 12 years of schooling (71.9%, 95% CI: 68.8; 75.0). Almost half (49.6%, 95% CI: 46.1; 53.0) were first time mothers and the mean age of the most recent infant was 59 days (range 3-150, 95% CI: 56.2; 61.9). The mean EPDS score was 7.8 (95% CI: 7.4; 8.2) and 24.3% (95% CI: 21.3; 27.2) women scored at or above the cut-off point of 12, which has been suggested in another study as appropriate for the Brazilian setting [23].

The preliminary one-factor CFA solution showed a poor fit. As shown in Table 1 (Model A), although the comparative indices did not indicate problems, the RMSEA did. The index's point estimate was 0.081 and the upper limit almost reached 0.1.

**Table 1:** Sequence of models concerning the Edinburgh Postnatal Depression Scale (EPDS): A) One-dimensional Confirmatory Factor Analysis; B) Exploratory/Confirmatory Factor Analysis (E/CFA); C) Three-factor Confirmatory Factor Analysis; D) Bifactor model: three specific factors plus a general (g) factor.

	Model A 1-factor CFA		Model B 3-factor - E/CFA				Model C 3-factor CFA				Model D Bifactor model				
				
	Factor 1		Factor 1	Factor 2	Factor 3		Factor 1	Factor 2	Factor 3		Factor 1	Factor 2	Factor 3	g-factor	
	***λ***_***i*(1)**_^**a**^	***δ***_***i***_^**b**^	***λ***_***i*(1)**_	***λ***_***i*(2)**_	***λ***_***i*(3)**_	***δ***_***i***_	***λ***_***i*(1)**_	***λ***_***i*(2)**_	***λ***_***i*(3)**_	***δ***_***i***_	***λ***_***i*(1)**_	***λ***_***i*(2)**_	***λ***_***i*(3)**_	***λ***_***i *(*G*)**_	***δ***_***i***_
i1	.74 (.70; .79)	.45	.81 (.47; 1.0)	.05 (-.23; .37)	-.02 (-.19; .16)	0.30	.82 (.77; .86)			.33	.48 (.25; .72)			.68 (.62; .74)	.30
i2	.71 (.66; .76)	.49	.83 (.66; 1.0)	-.03 (-.15; .1)	.01 (-.18; .21)	0.33	.78 (.73; .83)			.39	.50 (.26; .74)			.65 (.59; .71)	.33
i6	.59 (.54; .65)	.64	.31 (.15; .48)	.28 (.04; .52)	.08 (-.16; .31)	.63	.65 (.59; .70)			.58	.14 (.04; .24)			.52 (.45; .59)	.63
i3	.53 (.47; .59)	.72	.00 (-.04; .04)	.68 (.39; .97)	-.07 (-.37; .23)	.61		.60 (.53; .67)		.64		.25 (.10; .40)		.51 (.45; .58)	.67
i4	.53 (.48; .59)	.715	-.07 (-.29; .15)	.67 (.40; .94)	.01 (-.18; .20)	.61		.61 (.55; .67)		.63		.46 (.19; .72)		.63 (.56; .70)	.53
i5	.64 (.58; .70)	.59	.11 (-.10; .33)	.61 (.40; .83)	.01 (-.14; .15)	.52		.74 (.68; .80)		.46		.31 (.13; .48)		.59 (.53; .65)	.50
i7	.82 (.77; .86)	.33	.01 (-.08; .10)	.145 (-.08; .37)	.70 (.51; .89)	.31			.84 (.80; .88)	.30			.17 (-.02; .35)	.81 (.75; .88)	.31
i8	.80 (.77; .84)	.35	.05 (-.09; .18)	.01 (-.04; .06)	.78 (.66; .90)	.32			.83 (.79; .87)	.31			.24 (.03; .46)	.78 (.72; .84)	.33
i9	.81 (.78; .85)	.34	-.01 (-.07; .05)	-.15 (-.46; .15)	.99 (.74; 1.00)	.24			.84 (.80; .88)	.30			.45 (.22; .68)	.77 (.71; .83)	.20
i10	.68 (.60; .76)	.54	.03 (-.18; .24)	.06 (-.27; .40)	.62 (.31; .93)	.52			.70 (.62; .78)	.51			.17 (-.06; .40)	.67 (.57; .77)	.52
*f1 ⇔ f2*^c^	- - -		.66 (.47; .86)				.74 (.67; .81)				0				
*f1 ⇔ f3*	- - -		.75 (.63; .87)				.80 (.76; .85)				0				
*f2 ⇔ f3*	- - -		.82 (.69; .94)				.81 (.74; .87)				0				
RMSEA ^d^	.081 (.071; .091)		.037 (.019; .053)				.037 (.024; .049)				.026 (.005; .041)				
CFI ^e^	.963		.996				.993				.997				
TLI ^f^	.953		.991				.990				.995				

**^a ^**Loadings (standardized). In brackets: 95% confidence intervals. **^b ^**Measurement errors (uniqueness). **^c ^**Factors correlation; in brackets: 95% confidence intervals. **^d ^**RMSEA = Root Mean Square Error of Approximation. in brackets: 90% confidence intervals. ^e ^CFI = Comparative Fit Index. **^f ^**TLI = Tucker-Lewis Index.

Four increasingly complex two- to four-factor E/CFA models were sequentially fitted. The 2-factor E/CFA model showed a RMSEA of 0.057 (90% CI: 0.045; 0.069). A formal comparison of the 3-factor E/CFA model with this simpler model yielded a *χ*^2 ^value for difference testing of 49.223, which is highly significant (*p *≪ 0.001 at 8 d.f.) and shows a fit improvement. Although the 4-factor E/CFA model indicated an even better fit -- RMSEA of 0.028 (90% CI: 0; 0.050) -- and tested significantly *vis-à-vis *the 3-factor model (*χ*^2 ^= 18.876; p = 0.0086 at 7 d.f.), loadings on the forth factor were overall low (i_1 _= 0.002, i_2 _= 0.016, i_3 _= 0.335, i_4 _= 0.005, i_5 _= -0.008, i_6 _= 0.516, i_7 _= 0.164, i_8 _= 0.017, i_9 _= -0.087 and i_10 _= 0.125). Having decided for a model with three factors, we then looked into any possible residual correlations as indicated by the Modification Indices. Two correlations were suggested (i4⇔i5 = 0.425 and i3⇔i6 = 0.196), but freely estimating them in tandem showed different estimates from those projected by the MIs (i4⇔i5 = 0.193 and i3⇔i6 = 0.119). Thus, a three-factor model without residual correlations was regarded the most parsimonious. Findings are summarized in Table 1 (Model B). Factor 1 (anhedonia) encompassed items 1, 2 and 6, although the latter item showed cross-loadings on *f2 *and respective loadings were below the others. Factor 2 (anxiety) included items 3 to 5, and factor 3 (depression) encompassed items 7 through 10. All the three factors were highly correlated. Model fit proved to be reasonably good.

A strict CFA model was then fitted to the three-factor solution suggested in the E/CFA, with item 6 placed on factor 1 (Table 1 Model C). RMSEA (0.037) suggested a reasonable fit and loadings were fairly high. Of real interest here were the evaluations of convergent and, especially, discriminant validity. As shown in Table 2, AVE was low for *f2 *(0.43), indicating that convergent validity was questionable for at least one factor in the three-factor solution. Regarding factor 3, the respective square root of AVE used as benchmarks for evaluating discriminant validity was at or slightly above the related correlation, yet with quite some overlap of the confidence intervals. However, the magnitude of  for factor 1 was below one of its related factor correlation (*ϕ*_1↔3_), and  for factor 2 was far below both correlations (*ϕ*_1↔2 _and *ϕ*_2↔3_). Overall, discriminant validity was beneath acceptable levels and results suggested that alternative options should be investigated.

**Table 2:** Average variance extracted (*ρ*_*ve*(.)_), square root of AVE  and factor correlations (*ϕ*_.↔._), by factor.^a^

**Factor 1**			
	*ρ*_*ve*(*f*1)_	.56	(.52; .61)
		.75	(.72; .79)
**Factor 2**			
	*ρ*_*ve*(*f*2)_	.43	(.37; .48)
		.65	(.61; .70)
**Factor 3**			
	*ρ*_*ve*(*f*3)_	.65	(.61; .69)
		.81	(.78; .83)
**Factor correlations**			
	*ϕ*_1↔2_	.74	(.67; .81)
	*ϕ*_1↔3_	.80	(.76; .85)
	*ϕ*_2↔3_	.81	(.74; .87)

**^a ^**Values are based on the three-factor CFA solution (Model C in Table 1) based on the evidence suggested in the 3-factor E/CFA (Model B in Table 1), namely, factor 1 comprised by items 1, 2 and 6; factor 2 by items 3-5; and factor 3 by items 7-10. In brackets: 95% confidence intervals obtained by bootstrap method (B = 1000).

In view of these results and acknowledging an adequate item-factor specification as conveyed by the interim E/CFA, a bifactor model was fitted to examine the existence of a general factor, yet still accounting for (conditional on) the three specific factors (Table 1, Model D). Model fit improved further -- RMSEA (0.026, 90% CI: 0.005; 0.041), CFI (0.997) and TFI (0.995). Formally evaluating the significance of each factor showed that *f1 *and *f2 *were highly significant; both *χ*^2 ^for difference testing between the bifactor model (Model D) and the nested models in turn removing the factors under scrutiny had *p *≪ 0.001. The significance of factor 3 was less marked (p = 0.042). Regardless, loadings on the specific factors were all low to moderate . In contrast, all g-factor loading were all fairly high, ranging from 0.51 to 0.81 . As expected, testing the significance of the g-factor by comparing Model D with the three-dimensional CFA (Model C) yielded *p *≪ 0.001.

The percentages of variance explained by items, factors, and that due to errors (uniqueness) are shown in Table 3. Factors were accountable for 73.1% of variance. Notably, the g-factor answers for 79.2% of the explained variance, i.e., almost 4 times more than the share of all three specific factors put together.

**Table 3:** Percentage of variance explained by the general and specific factors, errors, and per items, according to the bifactor model (Model D)

Item	**Specific factors**			**g-factor**	**Error**
	1	2	3		
i1	3.0			6.0	1.2
i2	3.2			5.4	1.4
i6	.2			4.5	5.1
i3		.8		3.5	5.7
i4		2.7		3.4	3.6
i5		1.2		5.2	3.3
i7			.37	8.5	1.3
i8			.77	7.9	1.4
i9			2.6	7.7	.5
i10			.38	5.8	3.4
Sub-total	6.4	4.7	4.1		
Total		15.2		57.9	26.9

Note: all values above are %.

**Figure 2:**
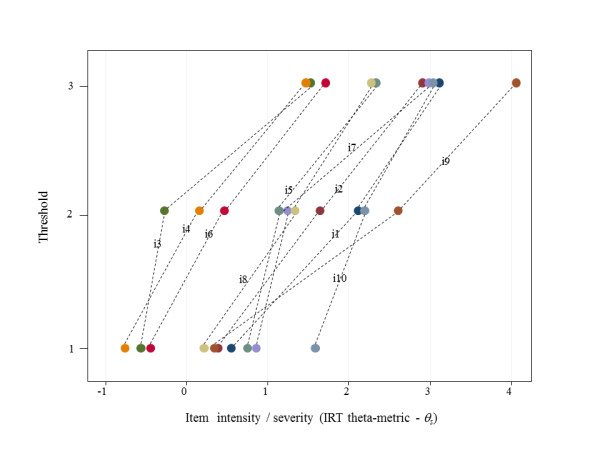
**Component items' thresholds pertaining to the four-level polychotomous items obtained in Model 1D**.

Figure 2 focuses primarily on the general factor as an encompassing scale mapping individuals along a continuous PPD latent dimension [20], and shows the *m *thresholds pertaining to the ten, four-level polytomous items. Although there are lines crossing-over item steps, there seemed to be an overall ordered gradient. The upward trends *vis-à-vis θ *for all items convey that women having a high level of PPD will display more symptoms and score increasingly higher item values.

Turning to the scrutiny of the raw scores as an applied representation of the latent factor scores, Table 4 provides the correlation matrix between them. Although the total raw score is fairly correlated to the factor-based specific scores, it correlates almost perfectly with the g-score (*r *= 0.974). The moderate correlations between the specific raw scores and the respective factor-based scores (*r_f1 _*= 0.654, *r_f2 _*= 0.784 and *r_f3 _*= 0.526) may be reflecting the particularities regarding the latter loadings as conveyed in Table 1, Model D -- e.g., small loadings of i6 on f1 (0.14), or i7 and i10 on f3 (0.17) --, as well as negative correlations between specific factor-scores.

**Table 4:** Correlation matrix between factor-based scores and respective raw scores.

	Factor score				Raw score			
		
	*g*	*f1*	*f2*	*f3*	total	*f1*	*f2*	*f3*
**Factor score**								
***g***	1.0							
***f1***	.200	1.0						
***f2***	.232	-.191	1.0					
***f3***	.291	-.311	-.273	1.0				
**Raw score**								
**total**	.974	.217	.325	.203	1.0			
***f1***	.791	.654	.032	-.050	.818	1.0		
***f2***	.738	-.029	.784	-.047	.801	.483	1.0	
***f3***	.895	-.020	.034	.526	.877	.597	.541	1.0

**Figure 3:**
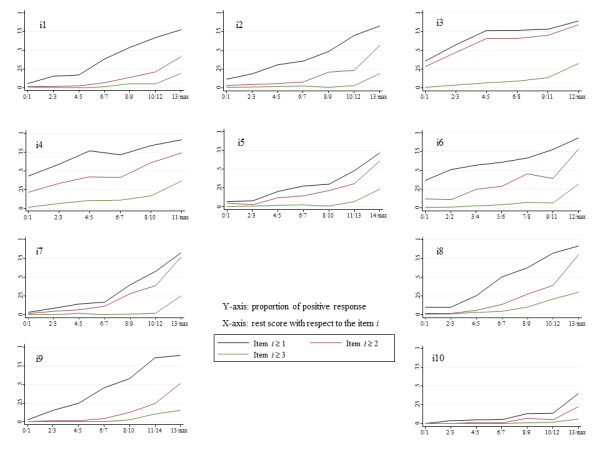
**Trace lines of items as functions of the restscore**.

The scale and items' Loevinger's H coefficients and the criteria for assessing single and double monotonicity assumptions are shown in Table 5. The numbers of absolute and relative violations *per *item (monotonicity and nonintersections) are also provided. All items' H*_Sj _*coefficients are above 0.3. The scale's overall scalability coefficient is also above this cut-off point (H = 0.4208). The monotonicity assumption was not rejected since no important violation occurred and all criteria were satisfied (Table 5, columns 2 and 3). This may be visualised graphically in Figure 3. None of the item-restscore traces showed intersections. This is not the case for inter-item ISRF nonintersections assessing the assumptions of double monotonicity as judged from the number of violations and related criteria shown in columns 4 and 5 of Table 5. Although six out of 10 criteria were less than 40 (i1, i3, i5, i6, i7 and i10) and for which violations can be merely ascribed to sampling variation, three items showed criteria between 40 and 80 (i2, i8 and i9) and one item (i4) exceeded 80. As a whole, the model followed by these data is therefore only partly double monotone.

**Table 5:** Items' and scale assessment of scalability (Loevinger's H coefficient), and checks for violation of monotonicity and double monotonicity assumptions (nonintersections of Item Step Response Functions).

		Monotonicity		Double monotonicity	
			
Item	H	**No. of violations **^**a**^	**Criteria **^**b**^	**No. of violations **^**a**^	**Criteria **^**b**^
i1	.4234	0		2 (.0010)	37
i2	.4086	0		3 (.0015)	46
i3	.3770	0		2 (.0010)	35
i4	.3605	1 (.0222)	13	12 (.0062)	82
i5	.4019	0		2 (.0010)	34
i6	.3880	1 (.0159)	11	4 (.0021)	38
i7	.4881	0		3 (.0015)	34
i8	.4673	0		9 (.0046)	70
i9	.4775	0		7 (.0036)	62
i10	.4201	0		0	
					
Total (scale)	.4208	4 (.0039)	---	---	---

**^a ^**See text for explanation. In brackets: proportion of violation *vis-à-vis *all possible violations regarding active pairs. See Molenaar, Sijtsma & Boer [52] and Hardouin, Bonnaud-Antignac & Sebille [51] for details.

**^b ^**See Hardouin, Bonnaud-Antignac & Sebille [51], equation 5 on page 39.

As judged by the preponderance of mostly positive properties described above, the sequence of increasing intensity/severity suggested by Figures 2 and 3 could therefore be described thus: i3 (self-blaming); i4 (anxious/worried for no reason); i6 (feeling of things getting on top); i8 (sad or miserable); i5 (scared/panicky for no reason); i7 (unhappy and difficulty in sleeping); i2 (no enjoyment to things); i1 (not able to laugh and see the funny side of things); i10 (thoughts of self-harming); and i9 (unhappy and crying).

## Discussion

As conveyed in the introduction, while the EPDS has had a huge following over the years, there has also been quite some disagreement about its dimensionality, especially in regards to the intricacies of its factor structure. Discussions as to the number of factors and the internal distribution of items have dominated the research program. Adding knowledge to the psychometric history of the EPDS, this study sought to step forward and examine some properties that had never been previously evaluated. As a positive start, we were able to corroborate the three-dimensional structure that has been previously reported in the literature [7-9,11,14,15]. More importantly, however, our study showed that, without further elaboration, this three-dimensional structure held rather poor factor-based discriminant validity. In principle, this would discourage using the EPDS as separate subscales.

Moving beyond a basic three-factor structure, results suggested that the EPDS was capable of distinguishing a general factor representing PPD. Moreover, in tandem with the related literature on mood and anxiety disorders [44], three specific factors could also be identified, although less conspicuously. While the three factors were significant on formal testing, specific loadings were low or moderate at the most. This contrasted with the g-factor loadings, which were sensibly higher. Overall, this picture goes in hand with the relatively low contribution of three specific factors to the total and explained variance, as opposed to the clear preponderance of a g-factor in the variance partition. As a side product, the currently proposed model (D) may also have unravelled a persistent problem found since the EPDS' conception, namely, that the consistent 'split' loadings found in several other settings regarding item 6 [10,14,15,53] were likely accountable to its contribution to a general factor that had not been properly specified.

It is thus laudable that the construct may be described as a single factor. The close connection between this general factor and the total raw score also suggests that the traditional practice of adding up raw component item scores to form an overall score is justifiable. To some extent, one may argue that the development process of the EPDS has 'come full circle' since the proposed 10-item score sum originally proposed by Cox et al. [2] is apparently tenable in practice as a first approach. Considering the other favourable properties that were detected (high correlation with the g-factor score, scalability and monotonicity), a total raw score may effectively be regarded as an empirical manifest of this general trait. In contrast, it is worth noting that the relatively weak correlations between specific-factor and related raw scores are indicative that separately using the EPDS sub-scales as raw scores in the postpartum period may not be so straightforward [6,18]. This is in line with the finding of Brouwers et al. [15] who identified that both anxiety and depressive symptoms were more accurately measured when using the total 10-item EPDS score.

Thus, a general PPD dimension that is seized through indicators (also) pertaining to three specific dimensions -- anhedonia, anxiety and depression -- may apparently be supported. Theoretically, anxiety and anhedonia, represented by their respective items and factors, would be only part of the spectrum of symptoms commonly observed in the presence of depression, not least in the period following childbirth. This proposition seems to agree with previous experimental and clinical observations that within a single episode of depression it is quite common for some symptoms of anxiety precede an overt depression symptomatology [54-56]. The helplessness/hopelessness perspective proposed by Alloy et al. [54] may clarify some features of the anxiety-depression co-morbidity. Accordingly, the intertwined relationship between anxiety and depressive symptoms is explained by noting that the anticipation of helplessness that comes with anxiety is likely also to trigger certain negative-outcome expectancies, and when all efforts to exert control eventually fail, overt depression follows. Nonetheless, the understanding that depressive and anxiety manifestations are sequential, alternate or concomitant facets of a common underlying process does also bring cogent theoretical support for the present empirical evidence, which is encouraging [57-60]. The meaningfulness of the actual crescendo in intensity and severity conveyed by mingled anhedonia, anxiety and depression-like symptoms (as outlined at the end of the results section) offers further support.

As many researchers in the field of measurement development and cross-cultural adaptation, we also take a universalistic approach and strive for psychometric consistency [61]. Yet, the answer as to whether a factorial structure will be repeatable in different socio-linguistic and cultural situations requires more research. New evidences of poor factor-based discriminant validity; followed by replicating and extending the models proposed here; along with theory-based external forms of construct validation studies using the EPDS as a general factor (or perhaps its 10-item raw score equivalent) should be a constructive way forward.

Yet, albeit still pending replication, it seems reasonable to reinforce the use of the full EPDS as a 10-item score. In applied research, the raw score may be indeed used as a first-approach 'mapper' of the underlying continuum. In a clinical perspective, the currently suggested cut-off points may be applied to help identify women who would benefit from in-depth psychiatric evaluation and ensuing follow up [4]. As a reminder, though, one should not ignore that identifying heterogeneous populations bearing primarily specific mood and anxiety disorders would require more complex instruments and that a definitive diagnosis of PPD cannot be established without a specialized assessment through more accurate means.

## Conclusion

This study attempted to take the debate on the dimensional structure of the EPDS a step forward. The present results suggest that, although a factorial analysis has again identified three factors, they lack factor-based discriminant validity and should not be used empirically as separate sub-scales. Nonetheless, albeit pending corroboration from further studies carried out in different settings, the total 10-item EPDS scale seems to be well suited as an empirical representation of a general factor representing PPD and its use in clinical practice and applied research is encouraged.

## Abbreviations

AVE: Average Variance Extracted; CFA: Confirmatory Factor Analysis; CFI: Comparative Fit Index; E/CFA: Exploratory/Confirmatory Factor Analysis; EFA: Exploratory Factor Analysis; EPC: Expected Parameter Changes; EPDS: Edinburgh Postnatal Depression Scale; MI: Modification Indices; PPD: Post-Partum Depression; RMSEA: Root Mean Square Error of Approximation; TLI: Tucker-Lewis Index; WLSMV: Weighted Least Squares Mean and Variance (adjusted estimator).

## Competing interests

The authors declare that they have no competing interests.

## Authors' contributions

Author MER managed funds for the Project, designed the study, wrote the protocol, supervised the data collection process, undertook the statistical analysis and collaborated in writing (first author) the final draft of the manuscript. Author CLM managed funds for the Project, designed the study, wrote the protocol, supervised the data collection process and collaborated in writing the final draft of the manuscript. Author ASDO collaborated in designing the study and writing the protocol, and was involved in coordinating the data collection process and assisted in writing the first draft of the manuscript. Author GL collaborated in designing the study and writing the protocol, was also involved in coordinating the data collection process, and assisted in writing up the manuscript. All authors read and approved the final manuscript.

## Pre-publication history

The pre-publication history for this paper can be accessed here:

http://www.biomedcentral.com/1471-2288/11/93/prepub
